# Efficient Removal 17-Estradiol by Graphene-Like Magnetic Sawdust Biochar: Preparation Condition and Adsorption Mechanism

**DOI:** 10.3390/ijerph17228377

**Published:** 2020-11-12

**Authors:** Yahui Zhou, Shaobo Liu, Yunguo Liu, Xiaofei Tan, Ni Liu, Jun Wen

**Affiliations:** 1 College of Environmental Science and Engineering, Hunan University, Changsha 410082, China; zhouyahui@hnu.edu.cn (Y.Z.); tanxf@hnu.edu.cn (X.T.); 2 Key Laboratory of Environmental Biology and Pollution Control (Hunan University), Ministry of Education, Changsha 410082, China; 3 College of Architecture and Art, Central South University, Changsha 410083, China; 4 School of Tourism Management, Hunan University of Technology and Business, Changsha 410205, China; meet_liuni@hnu.edu.cn; 5 College of Agriculture, Guangxi University, Nanning 530005, China; wenjun8852@126.com

**Keywords:** potassium ferrate, preparation, adsorption mechanism, carbonization, graphitization, water environment

## Abstract

The occurrence of environmental endocrine disrupting chemicals (EDCs) in aquatic environments has caused extensive concern. Graphene-like magnetic sawdust biochar was synthesized using potassium ferrate (K_2_FeO_4_) to make activated sawdust biochar and applied for the removal of 17-estradiol (E2). The characterization showed that the surface morphology of five graphene-like magnetic sawdust biochars prepared with different preparation conditions were quite different. The specific surface area and pore structure increased with the increment of K_2_FeO_4_ addition. The results have shown that graphene-like magnetic sawdust biochar (1:1/900 °C) had the best removal on E2. The experimental results indicated that pseudo-first-order kinetic model and the Langmuir model could describe the adsorption process well, in which the equilibrium adsorption capacity (*q*_e,1_) of 1:1/900 °C were 59.18 mg·g^−1^ obtained from pseudo-first-order kinetic model and the maximum adsorption capacity (*q*_max_) of 1:1/900 °C were 133.45 mg·g^−1^ obtained from Langmuir model at 298K. At the same time, lower temperatures, the presence of humic acid (HA), and the presence of NaCl could be regulated to change the adsorption reaction in order to remove E2. Adsorption capacity was decreased with the increase of solution pH because pH value not only changed the surface charge of graphene-like magnetic sawdust biochar, but also affected the E2 in the water. The possible adsorption mechanism for E2 adsorption on graphene-like magnetic sawdust biochar was multifaceted, involving chemical adsorption and physical absorption, such as H-bonding, π-π interactions, micropore filling effects, and electrostatic interaction. To sum up, graphene-like magnetic sawdust biochar was found to be a promising absorbent for E2 removal from water.

## 1. Introduction

Environmental estrogen, known as endocrine disrupting chemicals (EDCs), is a typical contaminant that could be frequently detected in the aquatic environment [1]. Environmental estrogen could significantly interfere with the normal reproductive function of aquatic organisms by affecting the synthesis, secretion, and transmission of the organism’s own estrogen, which could highly undermine the normal endocrine function of the human body and animal body [2,3]. E2 is considered to have the most potentially influential estrogens on endocrine disruption and can result in the feminization of males at extremely low concentrations of only 1 ng·L^−1^ in water [4,5]. Male trout exposed to E2 at levels as low as 1 ng·L^−1^ for five days are likely to be feminized and female specific proteins were produced in the blood of male mice when they are exposed to E2 at levels more than 5 ng·L^−1^ for five weeks [6]. E2 was found to be the main contributor to the estrogenic activities in most river waters because of its natural origin and high estrogenic potency [6].

Up to now, various methods have been explored to remove E2 from water, such as adsorption [1,2], photo-catalytic degradation [7,8], biodegradation [9], and advanced oxidation processes (AOPs) [10]. Recent researches have investigated AOPs based on sulfate radicals (SO_4_^2−^) [11] and based on highly reactive hydroxyl radicals (·OH) [12] for aqueous organic pollution degradation. Compared with AOPs, the adsorption method should be more economic for removing organic pollution at low concentrations [12]. Moreover, the adsorption technique also has the advantages of high efficiency, being renewable, and flexible operation [13]. Yin [4] has reported that the activated magnetic biochars (AMBCs) could be used as an effective adsorbent for removing E2 (the highest capacity 153.2 mg·g^−1^) by one-step synthesis. Mohammad [14] has researched that functionalized biochar with enhanced functional groups, specific surface area, and meso- and macro-pores prepared could be successfully applied for the removal of six phenolic EDCs. Biochar has been universally employed as a functional material for adsorption known for its large specific surface area, high porosity, and high physicochemical stability [15,16]. Agricultural and forestry biomass wastes, such as rice husk, sugarcane bagasse, wheat stem, wood chips, etc., are the raw materials for biochar production [15,17]. Furthermore, biochars produced by the waste biomass not only remove pollutants from the environment, but also solve environmental issues like the accumulation of solid wastes.

There are several methods for the modification of the biochar surface, including physical or chemical activation, steam activation, and coating for the contaminants’ removal from wastewater [18]. Among them, chemical modifications are generally used, which include acid and alkali treatment [19] and surface oxidation with oxidizers [20,21]. Some researchers reported that activated biochar had a relatively low electronic separated rate [11]. Therefore, the magnetic properties of activated biochar should be improved to enhance its adsorption performance as well as reusability. Gong et al. [22] synthesized highly porous graphitic biochar using K_2_FeO_4_ as an activator, which possessed a positive electrochemical performance as electrode materials for supercapacitors. Zhou et al. stated that K_2_FeO_4_ was a promising reagent because it could not only improve the pore structure of the material, but also generate new active sites, such as FeO_4_^2−^ and Fe_2_O_3_ [23]. In addition, the addition of the reagent K_2_FeO_4_ could load the iron oxide compound on the surface of the material, which provided convenience for the magnetic ionization technology, and at the same time overcame the disadvantages of the difficulty of collecting biochar after adsorption, making the recovery of biochar a reality.

Sawdust is an abundant biomass waste resource everywhere and sawdust biochar has great application potential in the removal of water environmental pollutants [24,25]. Preparing sawdust biochar adsorbent and applying it to the removal of pollutants in wastewater presents double effects in turning waste into treasure and improving the environment. K_2_FeO_4_ could fulfil the synchronous carbonization and graphitization for converting biomass to porous graphitic carbon biochar, which have been used as advanced electrode materials [22,26], catalytic degradation materials [11], and adsorption materials [18]. Based on previous researches, we prepared using K_2_FeO_4_ to produce graphitic biomass carbon (GBC) by the simultaneous carbonization and graphitization of sawdust biochar. K_2_FeO_4_ was utilized as graphitization catalyst (Fe), activating agent (KOH) and magnetizing agent (Fe) [11]. The synthetic process was done without the addition of any toxic substances, following the principles of green chemistry.

The main research objectives of this study are: (1) to produce graphene-like sawdust biochar with different pyrolysis temperatures and different K_2_FeO_4_ dosage; (2) to analyze the surface characteristics of the adsorbent with multiple characterization methods, such as scanning electron microscope (SEM), transmission electron microscope (TEM), X-ray diffraction (XRD), and X-ray photoelectron spectroscopy (XPS); (3) to explore the adsorption performance of graphene-like sawdust biochar on E2; (4) to discuss the possible adsorption mechanism of graphene-like sawdust biochar removal E2 from water.

## 2. **Materials and Methods**

### 2.1. Reagents

E2 (purity 98.0%, C_18_H_24_O_2_) was purchased from Sigma–Aldrich. K_2_FeO_4_, HA, and NaCl were obtained from Shanghai chemical corporation. Other chemicals including sodium hydroxide (NaOH), hydrochloric acid (HCl) and pH buffer solution (potassium hydrogen phthalate, mixed phosphate and bora), used to adjust the solution pH, were purchased from Shanghai Chemical Corp. Deionized water was obtained from Millipore Milli-Q water purification system. All reagents used were of analytical grade or higher and prepared with ultra-pure water.

### 2.2. Preparation of Sawdust Biochar

The feedstock of biochar used in this work was sawdust, which was collected from Yiyang farm, Hunan province, China. The sawdust was washed repeatedly with deionized water to remove impurities and dried at 65 °C in an oven for 24 h. Subsequently, the sawdust biomass was crushed and sifted through a 0.145-mm sieve. The pre-treated sawdust samples were then pyrolyzed in a tube furnace at 500 °C for 2 h under a nitrogen atmosphere with a heating rate of 7 °C min^−^^1^. The obtained black powders were washed with deionized water, then dried in the oven at 65 °C for 24 h. The black powders were finally ground to store in a sealed pocket and notated as BC.

### 2.3. Preparation of GBC

Several samples with different mass ratios (BC: K_2_FeO_4_) and annealed temperature (800 °C, 900 °C, 1000 °C) were prepared to explore the excellent preparation conditions. 2.0 g BC was dispersed in aqueous 0.1 M K_2_FeO_4_ solution whose volume was 20 mL, 50 mL, and 100 mL, respectively with continuous stirring for 12 h, and then dried at 65 °C to obtain. The mass ratios of BC and K_2_FeO_4_ were 1:0.2, 1:0.5 and 1:1 respectively. Subsequently, the solid mixture (1:0.2, 1:0.5, and 1:1) were heat-treated in a tube furnace at 900 °C for 2 h under a nitrogen atmosphere with a heating rate of 7 °C min^−1^. The mass ratio of 1:1 was heat-treated at a constant annealed temperature (800, 900, and 1000 °C) for 2 h under a nitrogen atmosphere with a heating rate of 7 °C min^−1^. The obtained samples were washed with deionized water, followed by drying at 65 °C. These resultant samples were notated as 1:0.2/900 °C, 1:0.5/900 °C, 1:1/900 °C, 1:1/800 °C, and 1:1/1000 °C (mass ratio (BC: K_2_FeO_4_)/ annealed temperature). The mass ratios (BC: K_2_FeO_4_) and annealed temperature are also in Table 1.

### 2.4. Characterization of Materials

The morphology and structure were characterized by field emission scanning electron microscope (SEM, Hitachi S4800, Tokyo, Japan). The graphitic structure was analyzed by high-resolution transmission electron microscopy (HRTEM, JEM 2000EX, JEOL, Tokyo, Japan). The surface chemical composition was determined by X-ray photoelectron spectroscopy (XPS, Thermo ESCALAB 250XI, Waltham, MA, USA). Raman spectrometer was performed on a Raman Microscope (LabRAM HR800, Paris, France) with YAG solid-state laser under wavelength of 532 nm. An X-Ray Diffractometer (XRD, D8 Advance Bruker, Germany) was used to study the crystallinity. The magnetic properties of samples were examined using a vibratory probe sample magnetometer (VSM-7300, Quantum design Lakeshore, San Diego, CA, USA). The specific surface areas were determined using the Brunauer–Emmett–Teller (BET, MicrotracBEL Corp, Shanghai, China).

### 2.5. Experimental Procedure

The effects of mass ratios (BC: K_2_FeO_4_) and annealed temperature on adsorbents properties were studied. Subsequently, the sample 1:1/900 °C was used for batch adsorption experiments based on the superbly adsorptive properties. In the beginning, three mass ratios (1:0.2/900 °C, 1:0.5/900 °C and 1:1/900 °C) and different annealed temperature (1:1/800 °C, 1:1/900 °C and 1:1/1000 °C) have been used for adsorption experiments. The experiments were performed in a 100 mL conical flask with 50 mL aqueous solution containing the fixed concentration of E2 and adsorbents (E2 = 6 mg·L^−1^, adsorbent = 0.005 g, T = 25 °C). More details on the selection of E2 and adsorbents concentrations were presented in Supplementary Figure S5. The bottles were shaken with a constant temperature shaker at 160 rpm. Sampling at different intervals, samples were filtered through 0.45-μm water membrane filter for analysis.

Batch adsorption experiments included kinetics adsorption experiments, isothermal adsorption experiments, pH effect, ionic strength effect, and OM effect on E2 adsorption which were conducted at 25 °C with a shaking speed of 160 rpm for 24 h in the vibrational chamber excluding kinetic adsorption experiments [11]. The solid/liquid ratio was 0.1 g/L in all batch experiments. The pH of experimental solutions was adjusted to 5 with NaOH and HCl except for the pH effect experiments. In the adsorption isotherm experiments, the solution with different E2 concentrations from 0.2 to 8mg·L^−1^ and adsorbents were added into conical flasks [11]. The pH effect experiments were conducted at pH from 3 to 10. To investigate the influence of coexisting organic matters on E2 removal, HA was selected as coexisting organic compounds. Different concentrations of NaCl (0–500 mmol·L^−1^) were used to investigate the effect of salinity on adsorption behavior [2]. For the reusability tests, the adsorbents were collected after using the first-time adsorption E2 in solution (20 mg·L^−1^) [18]. The desorption was carried out with the collected adsorbents. Firstly, the collected adsorbents were rinsed with 4 wt % NaOH solution and then the collected adsorbents were transferred into 50 mL absolute ethyl alcohol, followed by drying at 60 °C for reuse. All experiments were conducted twice, and the average value was adopted.

E2 concentration was determined using an F-4500 fluorescence spectrophotometer (Hitachi, Tokyo, Japan). The emission source is a 450 w xenon lamp which collected the emission matrix spectra of 5 nm in the excitation range of 200–400 nm and 300–500 nm, respectively. Fluorescence intensity of E2 was measured at E_x_/E_m_ = 280 nm/310 nm [27]. The data fitting model is provided in the Supplementary (Adsorption models).

## 3. Results and Discussion

### 3.1. Materials Characterization

**Figure 1:**
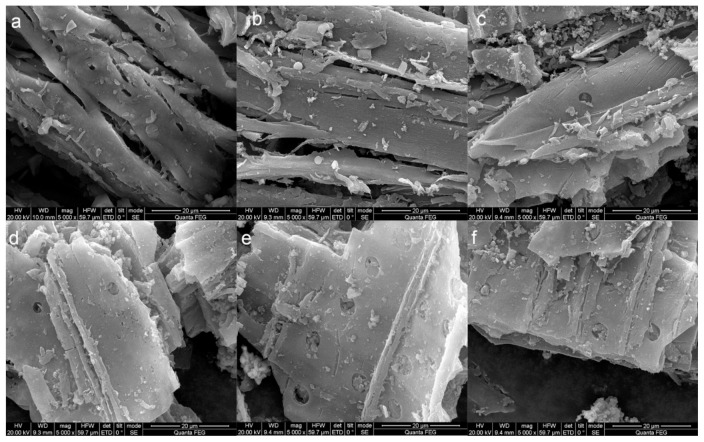
SEM images of the carbonaceous materials: (**a**) BC; (**b**) 1:0.2/900 °C; (**c**) 1:0.5/900 °C; (**d**) 1:1/800 °C; (**e**) 1:1/900 °C and (**f**) 1:1/1000 °C.

Surface morphology and microporous structure of carbonaceous materials were characterized by SEM. The surface of the BC was relatively smooth (Figure 1a). With the increasing of K_2_FeO_4_, the surface of GBC became more rough and uneven (Figure 1b–d). Moreover, a large number of regular pores were found in the Figure 1d–f, which further indicated that K_2_FeO_4_ played an important role in the formation of new micropores during the synchronous carbonization and graphitization processes. The abundant pores could offer more adsorption sites [28]. During the synthesis process, K_2_FeO_4_ was used as the activating agent. At a lower temperature, the activation began and formed K_2_CO_3_. Whereafter, K_2_CO_3_ started to decompose into K_2_O and CO_2_ with the high temperature (above 700 °C) [22]. Additionally, the produced CO_2_ and K_2_O could further react with carbon over 700 °C to K. During physical and chemical activations, the carbon lattices expanded irreversibly and resulted in many micropores. Since these chemical reactions occurred at over 700 °C, the specific surface area was mainly related to the addition ratio of the K_2_FeO_4_ [29]. More micropores are shown in Figure 1d–f than Figure 1b–d, so high dosage K_2_FeO_4_ made the microporous structure more powerful.

The N2 adsorption-desorption isotherms and mesoporous size distribution of carbonaceous materials were presented in Supplementary Figure S1. The BC adsorption-desorption isotherm of as-prepared sample was identified as type V and the five graphene-like magnetic sawdust biochar as identified as type IV with a hysteresis loop [30], which demonstrated the existence of mesoporous structure of adsorbent [31]. The pore-size distribution plot was presented in Supplementary Figure S2 and the pore-size distribution of carbonaceous materials appeared in a wide range 1.2–137.3 nm. The average pore diameter was around 1.2–1.5 nm. The presence of mesopores would be beneficial for adsorption in terms of the accessibility of the adsorbent to the active sites for adsorption reactions [32]. Moreover, the materials (1:1/800 °C, 1:1/900 °C and 1:1/1000 °C) exhibited a large surface area of 538.66 to 811.281 m^2^·g^−1^. This indicated high BET surface area and total pore volume of 1:1/900 °C in the Table 2. In contrast, the original BC exhibited a negligible BET surface area of 24.418 m^2^·g^−1^ and a tiny total pore volume of 0.020 cc/g. Nevertheless, the BET surface area was corresponding increase with K_2_FeO_4_ activation of the materials (1:0.2/900 °C and 1:0.5/900 °C). Definitively, the sample 1:1/900 °C demonstrated a powerfully abundant porous structure. The large accessible surface area and its suitable pore size distribution are beneficial for adsorption [33].

**Figure 2:**
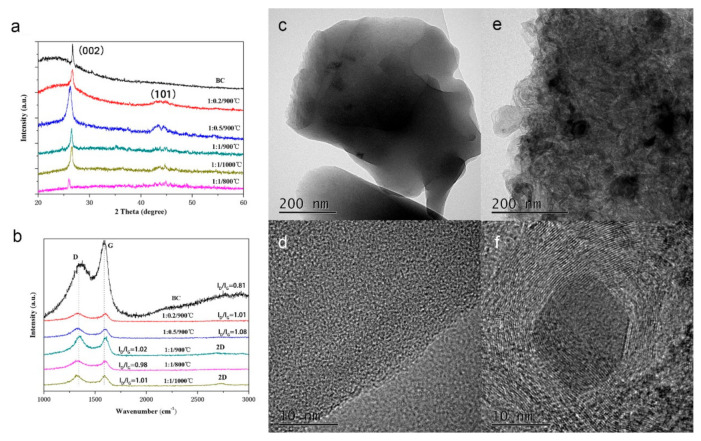
(**a**) XRD patterns and (**b**) Raman spectra of different samples; (**c**) TEM and (**d**) HRTEM image of the sample BC; (**e**) TEM and (**f**) HRTEM image of the sample 1:1/900 °C.

The crystallization and graphitization of the samples were observed by XRD and Raman. The XRD patterns were shown in Figure 2a. Only a peak of graphite (002) could be found on BC. The XRD patterns of GBC displayed two diffraction peaks approximately at 2θ values of 26.6 and 43.5, which were assigned to typical (002) plan and (101) reflections of graphitic carbon (JCPDS no. 41-1487) [11]. Thus, the XRD revealed the existence of crystalline structures on the samples after treated with K_2_FeO_4_ and annealed with high temperature. The graphitization was testified by Raman spectra (Figure 2b). All samples showed both the D band and G band at about 1346 cm^−1^ and 1590 cm^−1^, respectively. The samples of 1:1/900 °C and 1:1/1000 °C presented an inconspicuous 2D band at around 2700 cm^−1^. Generally, the D band is connected with disordered samples or graphene edges, while the G band is the phonon mode in-plane vibration of sp^2^-bonded carbon atoms [22]. The 2D band presents two phonon lattice vibration. The ratio of D band to G band (I_D_/I_G_) is generally used to reflect graphitization of samples. The I_D_/I_G_ value of BC, 1:0.2/900 °C, 1:0.5/900 °C, 1:1/900 °C, 1:1/800 °C and 1:1/1000 °C were 0.81, 1.01, 1.08, 1.02, 0.98, and 1.01, respectively. The increasing of the I_D_/I_G_ value indicated that the graphitization of the samples has been shown via the K_2_FeO_4_ excitation and high-temperature calcination. The 2D band of the samples 1:1/900 °C and 1:1/1000 °C also verified the formation of few-layered graphene rather than single-layer graphene [32,34,35]. Thus, the Raman spectra revealed the existence of graphitic structures on the samples after treated with K_2_FeO_4_ and annealed with high temperature. Moreover, the higher graphitization temperature was also beneficial to graphitization [29].

The TEM images of BC and 1:1/900 °C have been compared in order to further understand the properties of the materials before and after modification. In Figure 2c,e, the sample BC showed an extremely compact structure but the sample 1:1/900 °C was a fluffy structure with granular distribution, also indicating porosities of the sample 1:1/900 °C [32]. Simultaneously, from the HRTEM images of 1:1/900 °C (Figure 2f), a lattice fringe spacing was observed, corresponding to the graphite (002) plane, but it cannot be found on the HRTEM images of BC (Figure 2d). Furthermore, the TEM image of the sample 1:1/900 demonstrated its continuous porous structure, and the HRTEM image showed its distinct lattice fringes [36].

**Figure 3:**
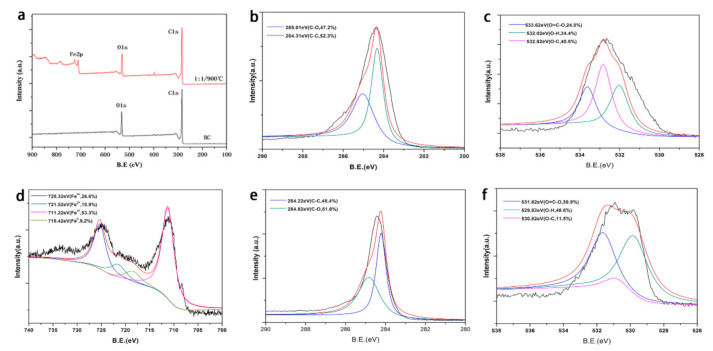
(**a**) XPS wide-scan; (**b**) C1s XPS spectra of BC; (**c**) O1s XPS spectra of BC; (**d**) Fe2p XPS spectra of 1:1/900 °C; (**e**) C1s XPS spectra of 1:1/900 °C; (**f**) O1s XPS spectra of 1:1/900 °C.

To get an insight into the surface chemical composition, the full survey scan XPS (Figure 3a) spectra showed the presence of C, O and Fe at the binding energy of 284.8 eV, 532.3 eV and 718.3 eV, respectively. The 1:1/900 °C had a distinct Fe peak, indicating successful synthesis of biochar with K_2_FeO_4_. The XPS peak deconvolution for C1s, O1s, and Fe2p spectra of samples was shown in Figure 3b–f. The XPS peak deconvolution for C1s, O1s had a slight difference between BC and 1:1/900 °C. Specifically, the peak of about 284.2 and 285.9 eV were assigned to C-C and C-O bonds, respectively. In Figure 3c, the peak of about 532.62 eV, 532.02 eV, and 532.82 eV were contributed to O=C-O, O-H and O-C bonds, respectively. The peaks of O1s existed slightly offset in Figure 3f. The surface of 1:1/900 °C produced more O=C-O which was conducive to the adsorption of E2. In Figure 3d, the Fe 2p spectrum could be deconvolved into four components. Two peaks at 711.22and 725.32 V could be attributable to Fe^3+^ species on the surface of 1:1/900 °C [37]. While the peaks at 718.42 and 721.52 eV could be assigned to Fe^0^ and Fe^2+^, respectively [11]. The proportion of Fe^0^ was only 9.2%, while the proportion of Fe^3+^ and Fe^2+^ was 79.9% and 10.9%, respectively. The 1:1/900 °C become magnetic biochar with the presence of Fe species and produced more oxygen-containing functional groups accompanied by the oxidation of Fe^3+^ species. From the above characterization results, it can be confirmed that the 1:1/900 °C sample was successfully synthesized by one-step process [11]. Supplementary Figure S3 displays the magnetic hysteresis curves of the sample 1:1 900 °C. The saturation magnetization of 1:1 900 °C from the magnetization curves were 0.059 emu·g^−1^ which suggested that the adsorbent was enough to achieve solid-liquid separation by a permanent magnet. These results reflected that biochar after modification had excellent magnetic properties and could be easily separated and recycled.

### 3.2. Adsorption Kinetics

**Figure 4:**
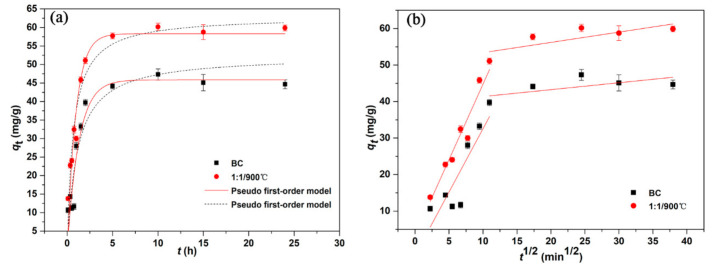
The adsorption kinetics study of E2 adsorption on BC and 1:1/900 °C (**a**) The pseudo first-order and pseudo second-order kinetics, (**b**) Intra-particle diffusion model.

As presented in Figure 4a, the E2 adsorption process could be divided into two stages, namely the fast adsorption stage and the slow adsorption stage [34]. E2 adsorption process was fast in the initial stage of 0~4 h and reached the equilibrium after 15 h. The rapid adsorption of E2 might be because of the electrostatic attraction between the negative charged E2 and the positive charged graphene-like magnetic biochar [38].

According to the correlation coefficient (*R*^2^) (Table 3), E2 adsorption kinetic data was well fitted by the pseudo-second-order and the pseudo-first-order kinetic models which demonstrated that the adsorption mechanism was mainly determined by chemisorption and physisorption [39]. When the adsorption was in equilibrium, the equilibrium adsorption capacities of BC and 1:1/900 °C were 45.71 mg·g^−1^ and 59.18 mg·g^−^^1^ obtained by the pseudo first-order model, respectively. The adsorption capacity of 1:1/900 °C was always higher than that of BC, which might be related to the abundant surface functional groups and large specific surface area [22]. Moreover, using the intraparticle diffusion model explored the E2 adsorption process. If the regression line of qt to t^1/2^ was a straight line and passes through the origin, the intra-particle diffusion was the main adsorption process [40,41]. In the Figure 4b, all the experimental data points were not on a straight line, which showed that intra-particle diffusion was not the only adsorption process. The first part of the line segment represented the diffusion process of E2 in the adsorbents membrane and the second part represented the diffusion process of E2 into the pores of the adsorbents. (That was diffusion within the particles). It could be seen that membrane diffusion and intra-particle diffusion worked together [2]. Therefore, the E2 adsorption process on graphene-like magnetic biochar was likely controlled by multiple processes.

### 3.3. Adsorption Isotherms

**Figure 5:**
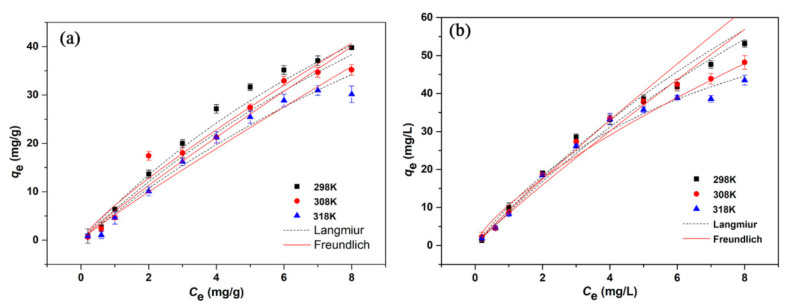
Isotherms of E2 adsorption by (**a**) BC and (**b**) 1:1/900 °C.

In Figure 5, the adsorption capacity of BC and 1:1/900 ℃ for E2 both increased with the initial concentration at three different temperatures, and their adsorption behavior was very similar.

However, the adsorption capacity of 1:1/900 °C was obviously better than that of BC. In the Table 4, the maximum adsorption capacity of 1:1/900 °C was 133.45 mg·g^−1^, while the maximum adsorption capacity of BC was 107.78 mg·g^−1^ at 298 K. The results indicated that low temperature was more conducive to the removal of E2 by graphene-like sawdust biochar. The relative parameters for two models were summarized in Table 4. The experimental data of E2 adsorption was fitted better with the Langmuir model by the comparison of the value of the correlation coefficients (*R*^2^), which suggested that the adsorption of E2 by graphene-like sawdust biochar occurred on a homogeneous surface were a monolayer adsorption process [34,39,42]. Referring to the significance of the parameter *n* of the Freundlich adsorption isotherm model (1 < *n* < 10 means the adsorption process is easy to proceed, *n* < 0.5 means the adsorption process is more difficult) [43], we could see that the adsorption of E2 by BC and 1:1/900 °C was easy to proceed at three different temperatures. With the *K*_f_ value related to the adsorption capacity of the Freundlich adsorption isotherm model (the larger the *K*_f_, the greater the adsorption capacity of the adsorbent) [44], it can be seen that low temperature was more helpful to the removal of E2 by BC and 1:1/900 °C.

### 3.4. Effect of Initial Solution pH on E2 Adsorption

**Figure 6:**
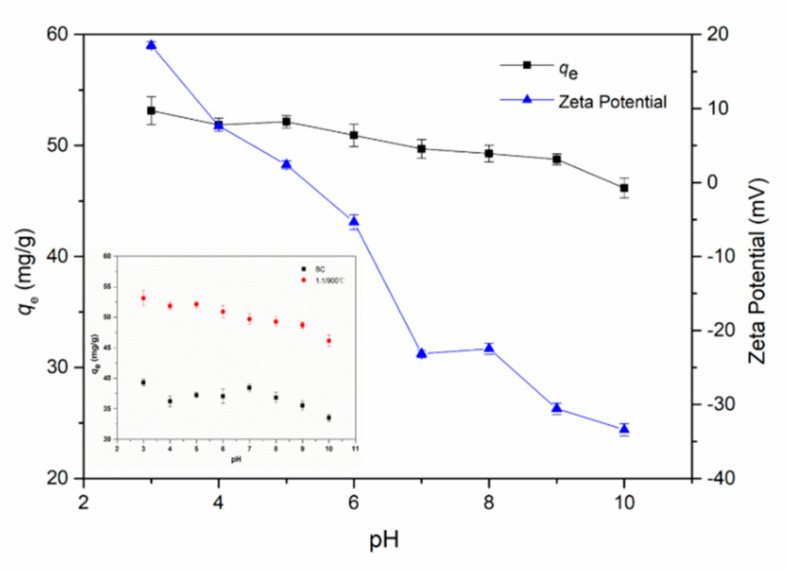
Effect of initial solution pH on E2 removal.

The pH value of the solution was crucial to the adsorption process, which not only changed the surface charge of biochar, but also affected the E2 in the water. In Figure 6, the effect of initial pH on E2 adsorption by BC and 1:1/900 °C was ranging from 3.0 to 10.0. The suitable pH value for graphene-like sawdust biochar removal E2 was about 5.0 and the adsorption capacities of 1:1/900 °C decreased when pH was ranging from 5.0 to 10.0. The phenomena might be caused by the change of the surface charge of biochar and the E2 speciation at different pH [35]. The zero-point zeta potentials of graphene-like sawdust biochar assessed was the pH = 5.1, which exhibited a relatively high adsorption capacity. Zeta potential results showed that graphene-like sawdust biochar on the surface was negatively charged within the scope of pH from 6 to 10. When the pH of the solution further increased to alkalinity, E2 had changed. Graphene-like sawdust biochar surface oxygen-containing groups further ionized, negative charge density increased, and electrostatic repulsion enhanced, leading to the decrease of adsorption capacity of E2 [24].

### 3.5. Effect of Ionic Strength

**Figure 7:**
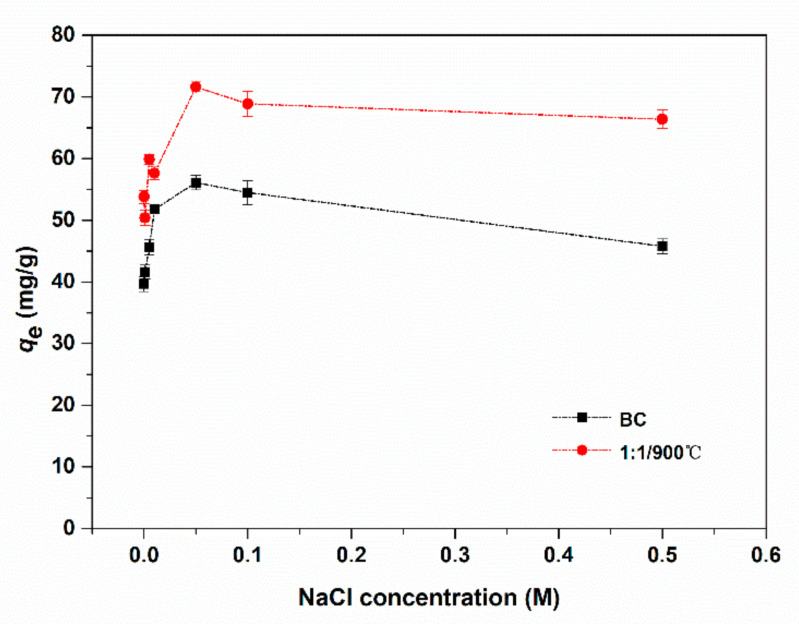
Effect of ionic strength on E2 removal by BC and 1:1/900 °C.

Furthermore, to investigate the effect of ionic strength on adsorption of E2 on graphene-like sawdust biochar, different concentrations of NaCl (0.0 M–0.6 M) were added to the E2 solution, owing to the popular existence of this ionic compound in wastewater. In Figure 7, the adsorbents BC and 1:1/900 °C had better adsorption capability on E2 in the presence of NaCl. When the NaCl concentration was less than 0.05 M, the removal E2 ability of BC and 1:1/900 °C also increased with the ionic strength increasing. When the NaCl concentration was greater than 0.05 M, the adsorbing capacity on E2 showed a downward trend, but the adsorption capacity of the two adsorbents for E2 was still higher than that without NaCl. There might be a potential explanation in this process according to some reports. Firstly, the activity coefficient of the hydrophobic organic compound increased as the ionic strength increased. However, the solubility of hydrophobic organic compound (that is the salting-out effect) decreased which was conducive to E2 adsorption [45]. Secondly, the repelling interaction between the adsorbents could be eliminated by penetration of the adsorbents surface and diffuse the electric double layer, and thereby forming a more compact polymer structure (i.e., squeezing-out), which was not conducive to E2 adsorption [2]. Therefore, an appropriate ionic strength like NaCl is conducive to the E2 adsorption, which could improve the E2 adsorption by changing it in the wastewater treatment.

### 3.6. Effect of Natural Organic Matter

**Figure 8:**
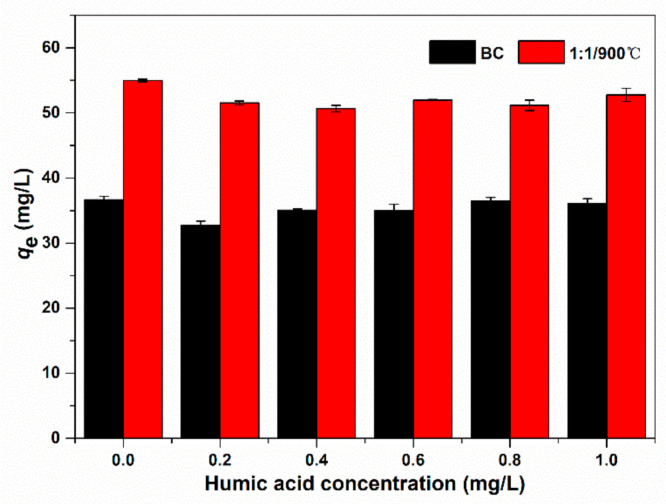
Effect of HA on E2 removal by BC and 1:1/900 °C.

Natural organic matters (NOMs) have been regarded as an important factor affecting the adsorption process since they widely existed in the natural environment [46]. They might interfere with the removal of targeted pollutants. HA is a representative component in NOMs. The influence of the NOMs on the adsorption removal E2 was investigated by HA addition. The adsorption capacity of BC and 1:1/900 °C on E2 decreased slightly from the Figure 8. Liu et al. [35] have researched that graphene-like magnetic lotus biochar had negligible effects on E2 adsorption with the increasing HA concentration, which suggested that HA might not directly compete for effective active sites on the surface of biochar and cause pore blockage, or HA might happen adsorption by the strong π-π interaction [38,47]. Therefore, when graphene-like sawdust biochar is used in wastewater treatment, the NOMs will not affect its adsorption performance.

### 3.7. Possible Mechanisms

Adsorbent recovery is an important consideration as it relates to the operating cost and the feasibility of its practical application. In Supplementary Figure S4, the adsorption capacity was decreased to 45.85 mg/g in the third cycle, but it remained at a high adsorption level in the third cycle, implying the stability of the 1:1/900 °C. It was superior to E2 adsorbents. Hence, 1:1/900 °C would be a potential adsorbent in the E2 removal in practical application due to its high adsorption performance and good regeneration performance.

Different characterizations and experiments were carried out to explore the possible E2 adsorption mechanisms onto 1:1/900 °C. In this work, the graphene-like magnetic structure of 1:1/900 °C have been observed by characterization. On the one hand, the sample 1:1/900 °C had a high surface area, abundant micropores, and high crystallinity properties. These surface structures were beneficial to E2 adsorption, while similar conclusions were also reported in [26,43,48]. Meanwhile, the XPS spectrum of 1:1/900 °C illustrated that its surface produced more O=C-O that was conducive to the adsorption of E2, which implied the involvement of oxygen-containing functional groups in the adsorption process. Some research has revealed that the contained oxygen functional groups could be improve the adsorption ability to E2 [18,38]. On the other hand, the graphene-like magnetic surface showed π-electron acceptor and E2 was a π-electron-rich contaminant due to the presence of aromatic rings [49]. Thus, π-π interaction played a significant function between the sample 1:1/900 °C and E2 contaminant.

## 4. Conclusions

(1) During the carbonization and graphitization process, when the mass ratio of BC to K_2_FeO_4_ was 1:1 and the second pyrolysis temperature was 900 °C, the graphene-like magnetic carbon sawdust biochar had the best adsorption capacity on E2.
(2) Different K_2_FeO_4_ dosages and pyrolysis temperatures had an important impact on the morphology and structure of the graphene-like magnetic carbon sawdust. The surface area of graphene-like magnetic sawdust biochar was affected by the K_2_FeO_4_ dosage. The more addition, the larger the specific surface area. However, under the condition that BC and K_2_FeO_4_ was 1:1, the surface area of graphene-like magnetic sawdust biochar showed a positively obvious result.
(3) The maximum adsorption capacity at 298 K was 133.45 mg·g^−1^ obtained from Langmuir model. Adsorption kinetics and adsorption isotherm indicated that the E2 adsorption by graphene-like sawdust biochar occurred on a homogeneous surface were a monolayer adsorption process and controlled by multiple processes.
(4) The pH, NaCl, and HA were crucial to the adsorption process because the adsorption capacity was affected by physical-chemical properties caused by the change of surface charge and the salting-out effect.
(5) The possible adsorption mechanism for E2 adsorption on graphene-like magnetic sawdust biochar was multifaceted, involving chemical adsorption and physical absorption, such as H-bonding, π-π interactions, micropore filling effects, and electrostatic interaction.

Based on the above conclusion, graphene-like magnetic sawdust biochar was found to be a promising absorbent for E2 removal from water as well as wastewater treatment, and the preparation method of graphene-like magnetic sawdust biochar was a green and feasible process. However, as a green technology, the economic viability might be a consideration and there should be some industrial viability of that process/product to be considered strictly.

## Figures and Tables

**Table 1:** The information about the graphitic biomass carbon.

Materials	Mass Ratios (BC: K_2_FeO_4_)	Annealed Temperature
1:0.2/900 °C	1:0.2	900 ℃
1:0.5/900 °C	1:0.5	900 ℃
1:1/900 °C	1:1	900 ℃
1:1/800 °C	1:1	800 ℃
1:1/1000 °C	1:1	1000 ℃

**Table 2:** Adsorption parameters of different materials.

Materials	Isotherms Type	BET Surface Area (m^2^/g)	Total Pore Volume (cc/g)	Pore Diameter (nm)
BC	V	24.418	0.020	1.253
1:0.2/900 °C	IV	85.663	0.064	1.345
1:0.5/900 °C	IV	266.087	0.255	1.391
1:1/900 °C	IV	811.281	0.526	1.351
1:1/800 °C	IV	538.660	0.275	1.348
1:1/1000 °C	IV	724.007	0.490	1.488

**Table 3:** Pseudo-first-order and pseudo-second-order equation model parameters for E2 adsorption on BC and 1:1/900 °C.

Adsorbents		BC	1:1/900 ℃
Pseudo first-order	K_1_ (1/min)	0.78	0.85
	q_e,1_(mg/g)	45.71	59.18
	R^2^	0.90	0.92
Pseudo-second-order	K_2_ (g/mg min)	0.02	0.02
	q_e,2_(mg/g)	51.48	63.79
	R^2^	0.87	0.91
Intra-particle diffusion	C_1_	−2.15	3.365
	K_1_	3.47	4.14
	R^2^	0.74	0.94
	C_2_	39.48	50.57
	K_2_	0.19	0.28
	R^2^	0.58	0.72

**Table 4:** Isotherm models parameters for E2 adsorption by BC and 1:1/900 °C.

Adsorbents	Temperatures	Langmuir			Freundlich		
		*q*_m_ (mg/g)	*K*_L_ (L/mg)	*R* ^2^	*K*_f_ (L/mg)	*n*	*R* ^2^
BC	298K	107.78 ± 4.23	0.076	0.995	7.44	1.20	0.981
	308K	104.63 ± 2.67	0.070	0.970	7.07	1.22	0.961
	318K	98.94 ± 3.21	0.064	0.988	5.98	1.18	0.981
1:1/900 ℃	298K	133.45 ± 5.32	0.08	0.996	10.55	1.28	0.990
	308K	103.60 ± 4.45	0.11	0.992	11.37	1.35	0.975
	318K	99.67 ± 3.53	0.10	0.987	9.38	1.28	0.973

## Supplementary Materials

The following are available online at https://www.mdpi.com/1660-4601/17/22/8377/s1, Figure S1: N_2_ adsorption-desorption isotherms of samples (a) BC; (b) 1:1/900 °C; (c) 1:1/800 °C; (d) 1:1/1000 °C; (e) 1:0.2/900 °C; (f) 1:0.5/900 °C; Figure S2: Pore size distributions of samples (a) BC; (b) 1:1/900 °C; (c) 1tabel:1/800 °C; (d) 1:1/1000 °C; (e) 1:0.2/900 °C; (f) 1:0.5/900 °C; Figure S3: The magnetization curves of 1:1 900 °C; Figure S4: Third consecutive desorption/adsorption cycles of 1:1/900 °C for E2 removal; Figure S5: The effect of modified conditions to E2 adsorption: (a) the different ratios of biochar to K_2_FeO_4_ (pyrolysis temperature: 900 °C, E2 = 6 mg L^−1^, m_(adsorbent)_ = 0.005 g, T = 25 °C); (b) the initial E2 concentration (m_(1:1/900 °C)_ = 0.005 g, T = 25 °C) (c) the pyrolysis temperature of adsorbents (the ratio of biochar to K_2_FeO_4_ was 1:1, E2 = 6 mg L^−1^, m_(adsorbent)_ = 0.005 g, T = 25 °C). References [50,51,52,53] are cited in the supplementary materials.

## References

[1] Jiang L., Liu Y.-G., Liu S.-B., Zeng G., Hu X., Hu X., Guo Z., Tan X., Wang L., Wu Z. (2017). Adsorption of Estrogen Contaminants by Graphene Nanomaterials under Natural Organic Matter Preloading: Comparison to Carbon Nanotube, Biochar, and Activated Carbon. Environ. Sci. Technol..

[2] Jiang L., Liu Y., Zeng G., Liu S., Hu X., Zhou L., Tan X., Liu N., Li M., Wen J. (2018). Adsorption of estrogen contaminants (17β-estradiol and 17α-ethynylestradiol) by graphene nanosheets from water: Effects of graphene characteristics and solution chemistry. Chem. Eng. J..

[3] Al-Khateeb L.A., Obaid A.Y., Asiri N.A., Salam M.A. (2014). Adsorption behavior of estrogenic compounds on carbon nanotubes from aqueous solutions: Kinetic and thermodynamic studies. J. Ind. Eng. Chem..

[4] Yin Z., Liu Y., Liu S., Jiang L., Tan X., Niu C.-G., Li M., Liu S., Tian S., Fang Y. (2018). Activated magnetic biochar by one-step synthesis: Enhanced adsorption and coadsorption for 17β-estradiol and copper. Sci. Total Environ..

[5] Dorabawila N., Gupta G. (2005). Endocrine disrupter—estradiol—in Chesapeake Bay tributaries. J. Hazard. Mater..

[6] Rao K., Lei B., Li N., Ma M., Wang Z. (2013). Determination of estrogens and estrogenic activities in water from three rivers in Tianjin, China. J. Environ. Sci..

[7] Qin C., Troya D., Shang C., Hildreth S., Helm R., Xia K. (2015). Surface catalyzed oxidative oligomerization of 17beta-estradiol by Fe(3+)-saturated montmorillonite. Environ. Sci. Technol..

[8] Wang W., Niu Q., Zeng G., Zhang C., Huang D., Shao B., Zhou C., Yang Y., Liu Y., Guo H. (2020). 1D porous tubular g-C3N4 capture black phosphorus quantum dots as 1D/0D metal-free photocatalysts for oxytetracycline hydrochloride degradation and hexavalent chromium reduction. Appl. Catal. B Environ..

[9] Bradley P.M., Writer J.H. (2014). Effect of Light on Biodegradation of Estrone, 17β-Estradiol, and 17α-Ethinylestradiol in Stream Sediment. JAWRA J. Am. Water Resour. Assoc..

[10] Liu Y., Cheng M., Liu Z., Niu C.-G., Zhong H., Chen M., Zhou C., Xiong W., Shao B., Song B. (2019). Heterogeneous Fenton-like catalyst for treatment of rhamnolipid-solubilized hexadecane wastewater. Chemosphere.

[11] Zhangab P., Tan X., Liu S.-B., Liu Y., Niu C.-G., Yeab S., Yinab Z., Huef X., Liuab N. (2019). Catalytic degradation of estrogen by persulfate activated with iron-doped graphitic biochar: Process variables effects and matrix effects. Chem. Eng. J..

[12] Peng G., Zhang M., Deng S., Shan D., He Q., Yu G. (2018). Adsorption and catalytic oxidation of pharmaceuticals by nitrogen-doped reduced graphene oxide/Fe_3_O_4_ nanocomposite. Chem. Eng. J..

[13] Ouyang J., Zhou L., Liu Z., Heng J.Y., Chen W. (2020). Biomass-derived activated carbons for the removal of pharmaceutical mircopollutants from wastewater: A review. Sep. Purif. Technol..

[14] Ahmed M.B., Zhou J.L., Ngo H.H., Johir A.H., Sornalingam K. (2018). Sorptive removal of phenolic endocrine disruptors by functionalized biochar: Competitive interaction mechanism, removal efficacy and application in wastewater. Chem. Eng. J..

[15] Tan X.-F., Liu S.-B., Liu Y.-G., Gu Y.-L., Zeng G.-M., Hu X., Wang X., Jiang L. (2017). Biochar as potential sustainable precursors for activated carbon production: Multiple applications in environmental protection and energy storage. Bioresour. Technol..

[16] Kwon G., Bhatnagar A., Wang H., Kwon E.E., Song H. (2020). A review of recent advancements in utilization of biomass and industrial wastes into engineered biochar. J. Hazard. Mater..

[17] Que W., Zhou Y.-H., Liu Y.-G., Wen J., Tan X.-F., Liu S.-J., Jiang L.-H. (2019). Appraising the effect of in-situ remediation of heavy metal contaminated sediment by biochar and activated carbon on Cu immobilization and microbial community. Ecol. Eng..

[18] Tam N.T.M., Liu Y., Bashir H., Yin Z., He Y., Zhou X. (2019). Efficient Removal of Diclofenac from Aqueous Solution by Potassium Ferrate-Activated Porous Graphitic Biochar: Ambient Condition Influences and Adsorption Mechanism. Int. J. Environ. Res. Public Health.

[19] Jin H., Capareda S., Chang Z., Gao J., Xu Y., Zhang J. (2014). Biochar pyrolytically produced from municipal solid wastes for aqueous As(V) removal: Adsorption property and its improvement with KOH activation. Bioresour. Technol..

[20] Wang Y., Wang X., Wang X., Liu M., Yang L., Wu Z., Xia S., Zhao J. (2012). Adsorption of Pb(II) in aqueous solutions by bamboo charcoal modified with KMnO4 via microwave irradiation. Colloids Surf. A Physicochem. Eng. Asp..

[21] Xue Y., Gao B., Yao Y., Inyang M., Zhang M., Zimmerman A.R., Ro K.S. (2012). Hydrogen peroxide modification enhances the ability of biochar (hydrochar) produced from hydrothermal carbonization of peanut hull to remove aqueous heavy metals: Batch and column tests. Chem. Eng. J..

[22] Gong Y., Li D., Luo C., Fu Q., Pan C. (2017). Highly porous graphitic biomass carbon as advanced electrode materials for supercapacitors. Green Chem..

[23] Zhou J., Liu Y., Pan J. (2017). Removal of elemental Mercury from flue gas using wheat straw chars modified by K2FeO4 reagent. Environ. Technol..

[24] Zhou Y., Liu X., Xiang Y., Wang P., Zhang J., Zhang F., Wei J., Luo L., Lei M., Tang L. (2017). Modification of biochar derived from sawdust and its application in removal of tetracycline and copper from aqueous solution: Adsorption mechanism and modelling. Bioresour. Technol..

[25] Zhu H., Tan X., Tan L., Chenac C., Alharbi N.S., Hayat T., Fang M., Wang X. (2018). Biochar Derived from Sawdust Embedded with Molybdenum Disulfide for Highly Selective Removal of Pb^2+^. ACS Appl. Nano Mater..

[26] Hu J., Li Z., Zhang A., Mao S., Jenkinson I.R., Tao W. (2020). Using a strong chemical oxidant, potassium ferrate (K_2_FeO_4_), in waste activated sludge treatment: A review. Environ. Res..

[27] Jiang L., Liu Y.-G., Zeng G.-M., Xiao F.-Y., Hu X., Hu X., Wang H., Li T.-T., Zhou L., Tan X.-F. (2016). Removal of 17β-estradiol by few-layered graphene oxide nanosheets from aqueous solutions: External influence and adsorption mechanism. Chem. Eng. J..

[28] Hu X., Wang W., Xie G., Wang H., Tan X., Jin Q., Zhou D., Zhao Y. (2018). Ternary assembly of g-C3N4/graphene oxide sheets /BiFeO3 heterojunction with enhanced photoreduction of Cr(VI) under visible-light irradiation. Chemosphere.

[29] Yao Y., Hu Y., Hu H., Chen L., Yu M., Gao M., Wang S. (2019). Metal-free catalysts of graphitic carbon nitride–covalent organic frameworks for efficient pollutant destruction in water. J. Colloid Interface Sci..

[30] Sing K.S.W. (1985). Pure and Applied Chemistry. Reporting physisorption data for gas solid systems with special reference to the determination of surface area and porosity. Pure Appl. Chem..

[31] Li Y., Shao J., Wang X., Deng Y., Yang H., Chen H. (2014). Characterization of Modified Biochars Derived from Bamboo Pyrolysis and Their Utilization for Target Component (Furfural) Adsorption. Energy Fuels.

[32] Kim M.-H., Tang J., Jang S.-J., Pol V.G., Roh K.C. (2019). Porous graphitic activated carbon sheets upcycled from starch-based packing peanuts for applications in ultracapacitors. J. Alloy. Compd..

[33] Maimaiti A., Deng S., Meng P., Wang W., Wang B., Huang J., Wang Y., Yu G. (2018). Competitive adsorption of perfluoroalkyl substances on anion exchange resins in simulated AFFF-impacted groundwater. Chem. Eng. J..

[34] Liu J., Jiang J., Aihemaiti A., Meng Y., Yang M., Xu Y., Gao Y., Zou Q., Chen X. (2019). Removal of phosphate from aqueous solution using MgO-modified magnetic biochar derived from anaerobic digestion residue. J. Environ. Manag..

[35] Liu N., Liu Y., Tan X., Li M., Liu S., Hu X., Zhang P., Dai M., Xu W., Wen J. (2020). Synthesis a graphene-like magnetic biochar by potassium ferrate for 17beta-estradiol removal: Effects of Al_2_O_3_ nanoparticles and microplastics. Sci. Total Environ..

[36] Fu H., Zhao P., Xu S., Cheng G., Li Z., Li Y., Li K., Ma S. (2019). Fabrication of Fe_3_O_4_ and graphitized porous biochar composites for activating peroxymonosulfate to degrade p-hydroxybenzoic acid: Insights on the mechanism. Chem. Eng. J..

[37] Gao Y., Li S., Li Y., Yao L., Zhang H. (2017). Accelerated photocatalytic degradation of organic pollutant over metal-organic framework MIL-53(Fe) under visible LED light mediated by persulfate. Appl. Catal. B Environ..

[38] Yin Z., Liu Y., Tan X., Jiang L., Zeng G., Liu S., Tian S., Liu S., Liu N., Li M. (2019). Adsorption of 17β-estradiol by a novel attapulgite/biochar nanocomposite: Characteristics and influencing factors. Process. Saf. Environ. Prot..

[39] Deng Y., Ansart R., Baeyens J., Zhang H. (2019). Flue Gas Desulphurization in Circulating Fluidized Beds. Energies.

[40] Svilović S., Rušić D., Basic A. (2010). Investigations of different kinetic models of copper ions sorption on zeolite 13X. Desalination.

[41] Zhou X., Shu L., Zhao H., Guo X., Wang X., Tao S., Xing B. (2012). Suspending Multi-Walled Carbon Nanotubes by Humic Acids from a Peat Soil. Environ. Sci. Technol..

[42] Liu J., Wang N., Zhang H., Baeyens J. (2019). Adsorption of Congo red dye on FexCo3-xO4 nanoparticles. J. Environ. Manag..

[43] Li M.-F., Liu Y., Zeng G.-M., Liu S.-B., Hu X., Shu D., Jiang L., Tan X.-F., Cai X.-X., Yan Z.-L. (2017). Tetracycline absorbed onto nitrilotriacetic acid-functionalized magnetic graphene oxide: Influencing factors and uptake mechanism. J. Colloid Interface Sci..

[44] Liu S.-J., Liu Y., Tan X.-F., Niu C.-G., Zhou Y.-H., Liu S.-B., Yin Z.-H., Jiang L.-H., Li M.-F., Wen J. (2018). The effect of several activated biochars on Cd immobilization and microbial community composition during in-situ remediation of heavy metal contaminated sediment. Chemosphere.

[45] Zhang S., Shao T., Bekaroglu S.S.K., Karanfil T. (2010). Adsorption of synthetic organic chemicals by carbon nanotubes: Effects of background solution chemistry. Water Res..

[46] Chowdhury R.R., Charpentier P.A., Ray M.B. (2011). Photodegradation of 17β-estradiol in aquatic solution under solar irradiation: Kinetics and influencing water parameters. J. Photochem. Photobiol. A Chem..

[47] Dong X., He L., Hu H., Liu N., Gao S., Piao Y. (2018). Removal of 17β-estradiol by using highly adsorptive magnetic biochar nanoparticles from aqueous solution. Chem. Eng. J..

[48] Zeng Z.-W., Tan X.-F., Liu Y.-G., Tian S.-R., Zeng G.-M., Jiang L.-H., Liu S.-B., Li J., Liu N., Yin Z.-H. (2018). Comprehensive Adsorption Studies of Doxycycline and Ciprofloxacin Antibiotics by Biochars Prepared at Different Temperatures. Front. Chem..

[49] Li Y., Wang Z., Xie X., Zhu J., Li R., Qin T. (2017). Removal of Norfloxacin from aqueous solution by clay-biochar composite prepared from potato stem and natural attapulgite. Colloids Surf. A Physicochem. Eng. Asp..

[50] Wang H., Liu Y.-G., Zeng G.-M., Hu X.-J., Hu X., Li T.-T., Li H.-Y., Wang Y.-Q., Jiang L.-H. (2014). Grafting of beta-cyclodextrin to magnetic graphene oxide via ethylenediamine and application for Cr(VI) removal. Carbohydr. Polym..

[51] Hu X., Wang J.-S., Liu Y.-G., Li X., Zeng G.-M., Bao Z.-L., Zeng X.-X., Chen A., Long F. (2011). Adsorption of chromium (VI) by ethylenediamine-modified cross-linked magnetic chitosan resin: Isotherms, kinetics and thermodynamics. J. Hazard. Mater..

[52] Fan T., Liu Y., Feng B., Zeng G., Yang C., Zhou M., Zhou H., Tan Z., Wang X. (2008). Biosorption of cadmium(II), zinc(II) and lead(II) by Penicillium simplicissimum: Isotherms, kinetics and thermodynamics. J. Hazard. Mater..

[53] Guo F.-Y., Liu Y.-G., Wang H., Zeng G.-M., Hu X.-J., Zheng B.-H., Li T.-T., Tan X.-F., Wang S.-F., Zhang M.-M. (2015). Adsorption behavior of Cr(vi) from aqueous solution onto magnetic graphene oxide functionalized with 1,2-diaminocyclohexanetetraacetic acid. RSC Adv..

