# Supplementary material for: Efficient Removal 17-Estradiol by Graphene-Like Magnetic Sawdust Biochar: Preparation Condition and Adsorption Mechanism
Source: Int J Environ Res Public Health. 2020 Nov 12;17(22):8377. doi: 10.3390/ijerph17228377 (PMC7696789; doi:10.3390/ijerph17228377)
Supplement: Supplementary file 1 [file ijerph-17-08377-s001.pdf]

Supplementary Materials: Efficient Removal 17-Estradiol by Graphene-Like Magnetic Sawdust Biochar: Preparation Condition and Adsorption Mechanism

Yahui Zhou [1,2], Shaobo Liu [3,*], Yunguo Liu [1,2,*], Xiaofei Tan [1,2], Ni Liu [4] and Jun Wen [5]

[1] College of Environmental Science and Engineering, Hunan University, Changsha 410082, China; zhouyahui@hnu.edu.cn (Y.Z.); tanxf@hnu.edu.cn (X.T.)

[2] Key Laboratory of Environmental Biology and Pollution Control (Hunan University), Ministry of Education, Changsha 410082, China

[3] School of Architecture and Art, Central South University, Changsha 410082, China

[4] School of Tourism Management, Hunan University of Technology and Business, Changsha 410205, China; meet_liuni@hnu.edu.cn

[5] College of Agriculture, Guangxi University, Nanning 530005, China; wenjun8852@126.com

* Correspondence: liushaobo23@aliyun.com (S.L.); hnuliuyunguo@gmail.com (Y.L.)

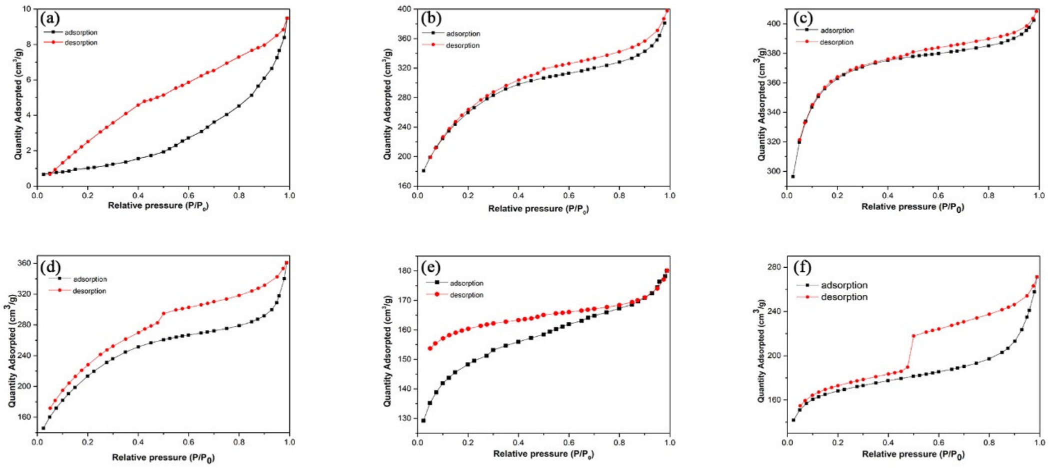

Figure S1. N_2 adsorption-desorption isotherms of samples (**a**) BC; (**b**) 1:1/900°C; (**c**) 1:1/800°C; (**d**) 1:1/1000°C; (**e**) 1:0.2/900°C; (**f**) 1:0.5/900°C.

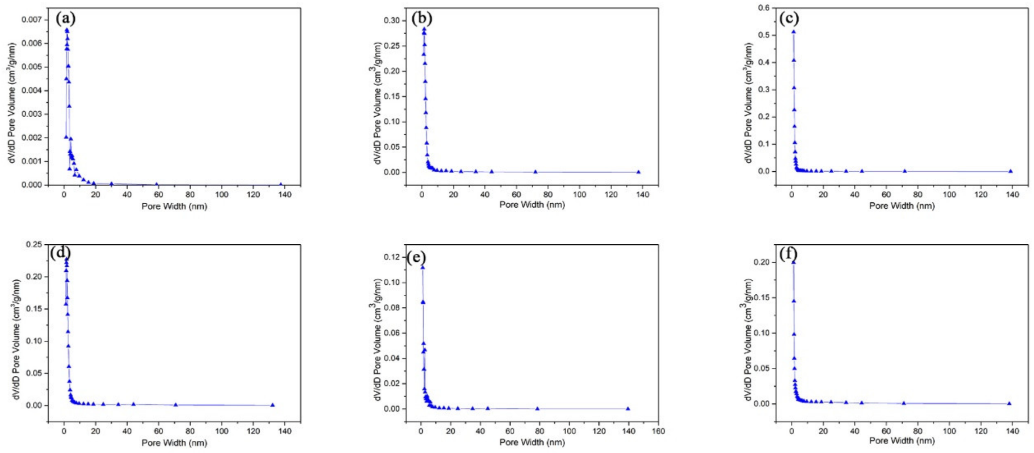

Figure S2. Pore size distributions of samples (**a**) BC; (**b**) 1:1/900°C; (**c**) 1:1/800°C; (**d**) 1:1/1000°C; (**e**) 1:0.2/900°C; (**f**) 1:0.5/900°C.

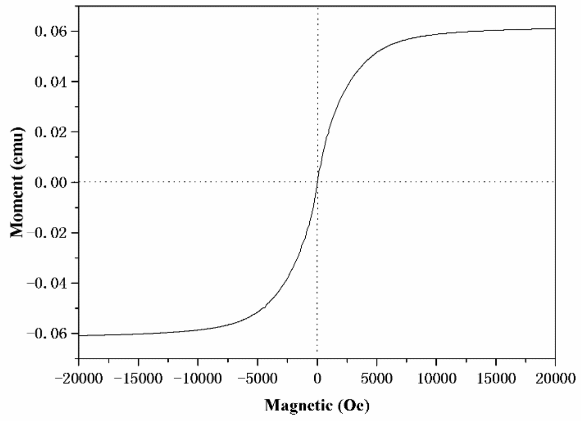

Figure S3. The magnetization curves of 1:1 900°C.

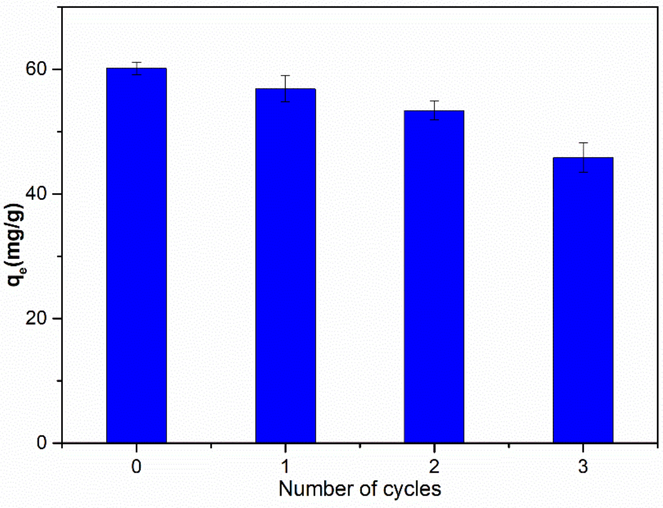

Figure S4. Third consecutive desorption/adsorption cycles of 1:1/900 ℃ for E2 removal.

1. Influence of modification conditions on adsorption

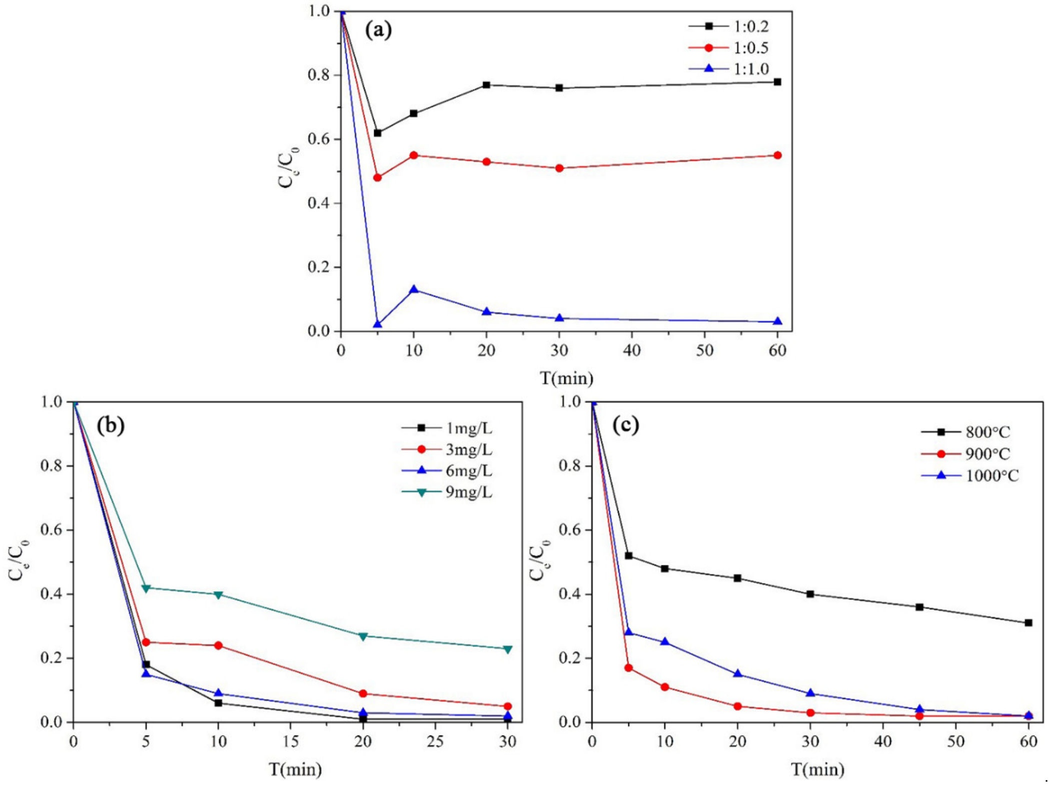

Figure S5. The effect of modified conditions to E2 adsorption: (**a**)the different ratios of biochar to K_2FeO_4 (pyrolysis temperature: 900℃, E2 = 6 mg L^{-1}, $m_{(adsorbent)}$ = 0.005g, T = 25 °C) ; (**b**)the initial E2 concentration ($m_{(1:1/900\ ℃)}$= 0.005g, T = 25 °C) (**c**)the pyrolysis temperature of adsorbents (the ratio of biochar to K_2FeO_4 was 1:1, E2 = 6 mg L^{-1}, $m_{(adsorbent)}$ = 0.005g, T = 25 °C).

In order to achieve the best adsorption effect of the biochar material, we have conducted an exploratory study on the preparation conditions of the biochar: the ratio of biochar to K_2FeO_4 and the pyrolysis temperature. The results are shown in Supplementary Figure S5a. It could be seen in 5.1(a) that the efficiency of removing E2 was greatly improved. during the 1 h reaction process, when the mass ratio of BC: K_2FeO_4 increased from 1:0.2 to 1:1. The adsorption rate increased from 87% to 99.5%. The initial E2 concentration was an important factor in the adsorption experiment. In the Supplementary Figure S5b, the removal rates of E2 were 99.8%, 98.5%, 99.5%, and 95.5%, respectively, at different concentrations of 1, 3, 6, and 9 mg L^{-1} when the adsorption was in equilibrium. The adsorption sites were easy to occupy due to the high concentration E2. The result indicated that it was beneficial to remove E2 when the initial E2 concentration was the 6 mg L^{-1}. Supplementary Figure S5c showed the effect of pyrolysis temperature on the removal of E2 under the same ratio of biochar to potassium ferrate (1:1). It could be observed that the entire adsorption process was divided into three stages. The first stage was fast adsorption (0–20 min), the second stage was slow adsorption (20–45 min), and the third stage was adsorption saturation stage (45–60 min). When the adsorption equilibrium was reached, the E2 adsorption rates of the samples at 800 °C, 900 °C, and 1000 °C were 94.8%, 99.6%, and 99.0%, respectively. Based on the above research, 1:1/900 °C graphitized biochar was chose for batch experiments and mechanism exploration with original biochar.

2. Adsorption models

2.1. Kinetic Models

The pseudo-first-order kinetic model is generally given as [50]:

$$\ln(q_{e,1} - q_t) = ln\, q_{e,1} - k_1 t \quad (1)$$

where k_1 (min^{-1}) stands for the first-order reaction rate equilibrium constant. $q_{e,1}$ and q_t are the adsorbed amount at equilibrium and at time t (mg g^{-1}), respectively.

The pseudo-second-order kinetic model is generally presented as [2]:

$$\frac{t}{q_t} = \frac{1}{k_2 q_{e,2}^2} + \frac{t}{q_{e,2}} \quad (2)$$

where k_2 (g mg^{-1} min^{-1}) stands for the first-order reaction rate equilibrium constant. $q_{e,2}$ and q_t denote the adsorbed amount at equilibrium and at time t (mg g^{-1}), respectively.

The intraparticle diffusion model is described as [51]:

$$q_t = k_p t^{0.5} + C \quad (3)$$

where k_p is a constant of intraparticle diffusion rate (mg/g $min^{0.5}$) and C can be determined from the intercept of linear fittings of q_t versus $t^{0.5}$.

2.2. Adsorption Isotherm Models

The Langmuir and Freundlich models were used to simulate the adsorption isotherms. The following expression illustrates the Langmuir equation [2,52]:

Langmuir：

$$q_e = \frac{q_{max} K_L C_e}{1 + K_L C_e} \quad (4)$$

where q_e and q_{max} are the adsorbed amount at equilibrium and the maximum adsorption capacity of the adsorbent (mg g^{-1}), respectively. C_e stands for the equilibrium solution phase concentration, and K_L denotes the Langmuir constant related to the energy of adsorption (L mg^{-1}).

The Freundlich equation is described by the following expression [53]:

Freundlich：

$$q_e = K_F C_e^{n} \quad (5)$$

where q_e and C_e are defined the same as provided in the Langmuir equation. K_F [(mg g^{-1}) (mg $L^{-1})^{-1}$n] stands for the adsorption affinity coefficient, while n is the exponential coefficient.
